# Infection prevention and control practice among home‐care nursing agencies in Japan: Secondary analysis of a nationwide cross‐sectional survey

**DOI:** 10.1111/ggi.14266

**Published:** 2021-09-01

**Authors:** Noriko Morioka, Masayo Kashiwagi

**Affiliations:** ^1^ Graduate School of Healthcare Sciences Tokyo Medical and Dental University Tokyo Japan

**Keywords:** cross‐sectional survey, home‐care nursing, infection prevention and control, Japan

## Abstract

**Aim:**

We describe the nationwide situation of infection prevention and control (IPC) practices among home‐visit nursing agencies and compare them by agency size to explore whether these practices are associated with the occurrence of infection.

**Methods:**

We conducted a secondary analysis using data from a cross‐sectional nationwide survey inspecting patient safety and IPC practices among nationwide home‐visit nursing agencies, from March to April 2020. Among 9978 agencies, 580 responded and 370 were incorporated in the analysis. The self‐administered questionnaire inquired about the IPC policy and administrative structure, education and training, adherence to standard precautions, and employee health programs. We described the adherence to IPC practice at the agency level and compared them by agency size using chi‐squared tests. Logistic regression analysis was performed to explore the associations between IPC practices and incidence of infection.

**Results:**

Adherence to IPC practices ranged from 19.2% to 92.4% and varied according to agency size. Less than 20% of agencies had instituted a committee for IPC and strictly used disposable aprons when changing patients' diapers. Instituting a committee for IPC (odds ratio 2.19, 95% confidence interval 1.11–4.34, *P* < 0.05) and training staff for infection prevention (odds ratio 1.67, 95% confidence interval 1.02–2.72, *P* < 0.01) were significantly associated with the incidence of infection, after adjusting for covariates.

**Conclusions:**

There are challenges in establishing IPC policies and administrative structures and adhering to standard precautions. Well‐organized agencies were found to be more likely to detect infections occurring over the past 3 months. **Geriatr Gerontol Int 2021; 21: 913–918**.

## Introduction

Globally, condition management of home‐care patients is a serious public health issue as the population subjected to complex diseases and medical devices is increasing rapidly in the community.^1^, ^2^ In particular, infection prevention and control (IPC) are crucial factors that enable long‐term home care. Approximately 5%–11.5% of patients using home healthcare services report infections,^3^, ^4^, ^5^ and 17% of all unplanned hospitalizations are caused by respiratory, urinary tract, intravenous catheter‐related and wound infections.^6^ Previous studies have recommended the following IPC practices at home‐care settings: adherence to published standards, compliance with standard precautions, including hand hygiene and utilization of personal prevention equipment (PPE) (e.g., surgical masks, gloves, gowns), provision of recommended immunizations, appropriateness of medical devices and capacity building of employees regarding IPC.^1^, ^2^, ^7^, ^8^, ^9^ Although the significance of IPC is well‐established, lack of a suitable structure and process of care for recommended IPC practices due to limited resources at home‐care settings has been identified. There exist many challenges in the compliance of home‐care nurses with IPC practices.^9^, ^10^, ^11^, ^12^, ^13^ Observation surveys reveal that almost half of home‐care nurses adhered to hand hygiene practices,^11^, ^13^ and about 20% of home‐care nurses reported receiving insufficient education to recognize infections among patients.^9^ Furthermore, the availability of IPC supplies, including alcohol‐based hand sanitizers, gloves and masks, training for employees, agency policies and procedures varied across home healthcare agencies.^10^ The study also reported that such agency‐level availability was associated with the adherence to IPC among home‐care nurses after adjusting for individual nurses and patients' home environment. Agency‐level factors are essential to ensure abidance to IPC practices among home‐care nurses.

Currently, Japan contains a super‐aged society with 28.4% of people over the age of 65 years.^14^ The government has established a community‐based integrated care system wherein the elderly can live independently in environments most familiar to them, even when requiring long‐term and medical care. Home‐care nursing services were introduced via long‐term care insurance and National Health Insurance.^15^, ^16^ The annual number of patients using home‐care nursing services is nearly 5 838 000 and increasing rapidly over the years.^17^ Moreover, patients undergoing medical treatments and relying on medical instruments have also been increasing.^18^ Past research signifies that 15% of home‐care patients experience some kind of infection in a year,^19^ and fever occurred at least once annually among one‐third of the participants.^20^ Although it has been specified that standard precaution is important, as it is in other countries,^21^ little is known about adherence to IPC practices at agency‐level home‐health services. Additionally, Japanese home‐care nursing agencies hire a low number of nurses, with half of the facilities employing less than four nursing staff members.^22^ Besides, the situation of IPC practice might differ due to agency size,^23^ with some agencies lacking resources related to infection prevention.

Thus, we aimed (i) to describe the nationwide situation of IPC practices among home‐care nursing agencies and compare those by agency size, and (ii) to explore whether such practices are associated with the occurrence of infection among patients who use home‐care nursing services.

## Methods

### Study design

We performed a secondary analysis using data from a cross‐sectional survey investigating patient safety and IPC practices among nationwide home‐care nursing agencies.^24^ The survey was conducted at the end of March 2020. The self‐administered questionnaires were mailed to 9979 home‐care nursing agencies deemed operational by the Information Publication System for Long‐term Care database of the Ministry of Health, Labour and Welfare.^25^ Currently, 10 884 home‐care nursing agencies operate in Japan according to the 2018 Survey of Institutions and Establishments for Long‐term Care.^26^ The database used in this study covered 91.7% of all such agencies. The self‐reported practices of nursing administrators for patient safety or IPC, and the number of adverse events and infections occurring in their agencies during the last 3 months were recorded. Questionnaire items were developed based on relevant guidelines^7^, ^27^ and literature review, and content validity was assessed by home‐care or patient safety management experts. Among 9979 agencies, 580 returned the questionnaires. After deleting incomplete responses, refusal to participate in the study (*n* = 84) and missing data (*n* = 126), 370 responses were included in the final analysis (i.e., a final response rate of 3.7%). Despite the relatively low response rate, the response rates by region were almost identical (Table S1). We could not mail the remaining letters due to the COVID‐19 outbreak in Japan that coincided with the study period.

### Measurements

#### Infection prevention and control practice

To assess IPC practice at a nursing agency level, we used (i) IPC policy and administrative structure, (ii) education and training, (iii) adherence to standard precautions, including hand hygiene and use of PPE, and (iv) employee health program as per relevant guidelines and previous studies.^8^, ^27^ For the IPC policy and administrative structure, we evaluated whether a manual for IPC existed (yes or no), whether a committee for IPC was established (yes or no), whether a representative was assigned for infection control (yes or no), and whether appropriate information was exchanged with other agencies regarding IPC (yes or no). As for education and training, the training of nursing administrators for IPC (yes or no) and training of staff for infection prevention (yes or no) were inquired into. Information regarding adherence to standard precautions such as evaluation of hand hygiene compliance among nursing staff (yes or no), provision of portable alcohol hand sanitizer to staff (yes or no), changing diapers with disposable gloves (always or not), and changing diapers with disposable aprons (always or not) was solicited. For the employee health program, information concerning monitoring the results of vaccination and antibody titer test for staff (yes or no) was requested.

#### Incidence of infection

The nursing administrator reported the number of infections detected in the last 3 months, which comprised respiratory, skin, soft tissues, urology and catheter‐related infections. The numbers of infections were self‐reported regardless of whether laboratory testing or diagnosis was conducted. We used the incidence of infection as a dichotomous variable (i.e., no infection or at least one infection).

#### Agency characteristics

For other characteristics related to agencies, we used years since establishment, agency ownership (i.e., healthcare corporation, profit, social welfare, or others), and agencies with a medical institution (yes or no). As nursing staff variables, we employed nurse managers' years of experience as a manager, the number of full‐time equivalent nurses, and nursing staff with advanced practice certification (i.e., certified nurse [CN] or certified nurse specialist [CNS]) (yes or no). All the fields of CN and CNS were included in advance practice certification in this study, because all CN and CNS training programs include patient safety and quality management. To appraise the patients' characteristics at the agency level, the number of patients in a month, acceptance of pediatric patients (yes or no), acceptance of patients at the terminal care stage (yes or no), percentage of patients with care need level ≥3, percentage of patients under medical treatment, such as patients with cancer, tracheostomy, self‐peritoneal perfusion, home oxygen therapy and central venous nutrition were utilized. The details are provided accordingly.^24^

### Statistical analyses

We described the percentage of IPC practices and the summary of agency characteristics. The quartile of full‐time equivalent nurses was calculated as the agency size. To compare IPC practices via agency size, we conducted chi‐squared tests. Univariate logistic regression models were applied to investigate the association between the incidence of infections and IPC practice. In the multivariate logistic models, independent variables were the 11 items of IPC practice, and selected variables (i.e., agency ownership, agency with medical institution, number of full‐time equivalent nurses, percentage of patients with care need level ≥3 and percentage of patients under medical treatment) whose univariate test had a *P*‐value of <0.25, along with all variables of known clinical importance, were included in the analysis as covariates. Statistical *P* < 0.05 was set as significant, and all analyses were performed using Stata version 16 (StataCorp. College Station, TX, USA).

### Ethical considerations

The protocol was approved by the Medical Research Ethics Committee of the Tokyo Medical and Dental University (No. M2019‐304). Participants signed an informed consent form in the questionnaire to participate in the study.

## Results

Table 1 presents the characteristics of the 370 home‐care nursing agencies included in the analysis. About 40% of the agencies owned profit organizations and medical institutions (i.e., a hospital or a clinic). The median (25–75 percentile) number of full‐time equivalent nurses was 4 (3–5.8). Of 370 agencies, 113 (30.5%) had experienced at least one or more infections during the last 3 months, and a total of 3247 infections were detected. The median (25–75 percentile) of number of infections among 370 agencies was 3 (0–9). The medians (25–75 percentile) of number of infections by the quartile of full‐time equivalent nurses (the 1st quartile group, the 2nd quartile group, the 3rd quartile group and the 4th quartile group) were 2 (0–7), 2 (0–6), 4 (0–12) and 5 (0–19), respectively.

**Table 1:** Characteristics of home‐care nursing agencies (*N* = 370)

Variables		
Years since establishment (median, 25–75 percentile)	6.6	3.1–19.9
Agency ownership (*n*, %)		
Healthcare corporation	111	30.0
Profit	159	43.0
Social welfare	80	21.6
Others	20	5.4
Agencies with a medical institution (*n*, %)		
No	234	63.2
Yes	136	36.8
Nurse managers' years of experience as a manager (median, 25–75 percentile)	3.0	1.0–5.0
Number of full‐time equivalent nurses (median, 25–75 percentile)	4.0	3.0–5.8
Percentage of nurses who worked <1 year (median, 25–75 percentile)	12.5	0.0–27.3
Nursing staff with advanced practice certification (*n*, %)		
No	319	86.2
Yes	51	13.8
Number of patients in a month (median, 25–75 percentile)	54.0	32.0–81.0
Accept pediatric patients (*n*, %)		
No	287	77.6
Yes	83	22.4
Accept patient at terminal care stage (*n*, %)		
No	267	72.2
Yes	103	27.8
Percentage of patients with care need level ≥3 (median, 25–75 percentile)	28.3	19.1–37.5
Percentage of patients who are under medical treatment (median, 25–75 percentile)	16.7	7.8–27.4

**Figure 1:**
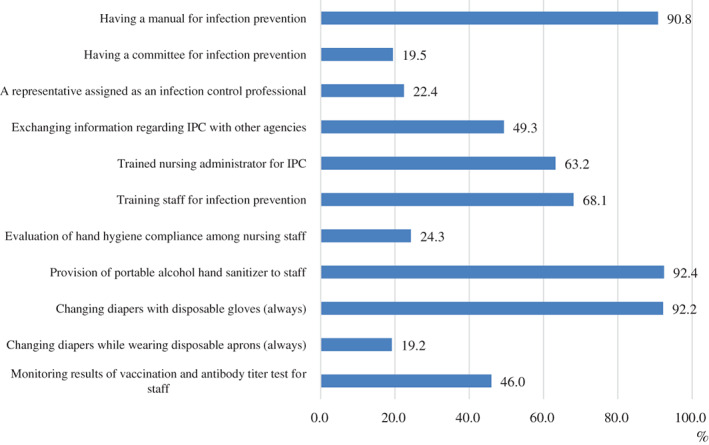
Infection prevention and control practice among home‐care nursing agencies (*N* = 370).

IPC practice percentages ranged from 19.2% to 92.4% (Fig. 1). Over 90% of agencies had a manual for IPC (90.8%), provided portable alcohol hand sanitizer to staff members (92.4%) and had used disposable gloves when changing patients' diapers (92.2%). Less than 20% of agencies had a committee for IPC (19.5%), and the staff always used disposable aprons when changing patients' diapers (19.2%). By stratifying agency size, larger agencies were more likely to adopt a manual for IPC (*P* = 0.040), institute a committee for IPC (*P* = 0.006) and conduct an antibody titer test for staff members (*P* = 0.030) (Table 2).

**Table 2:** Comparing adherence to IPC practice according to agency size (*N* = 370)

	Total		According to the size of home‐care nursing agencies								
			1st quartile		2nd quartile		3rd quartile		4th quartile		
	*n*	%	*n*	%	*n*	%	*n*	%	*n*	%	*P*‐value
Having a manual for infection prevention											
No	34	100.0	15	44.1	2	5.9	10	29.4	7	20.6	0.040
Yes	336	100.0	93	27.7	82	24.4	77	22.9	84	25.0	
Having a committee for infection prevention											
No	298	100	89	29.9	72	24.2	75	25.2	62	20.8	0.006
Yes	72	100	19	26.4	12	16.7	12	16.7	29	40.3	
A representative assigned as an infection control professional											
No	287	100	88	30.7	65	22.7	69	24.0	65	22.7	0.380
Yes	83	100	20	24.1	19	22.9	18	21.7	26	31.3	
Exchanging information regarding IPC with other agencies											
No	186	100	56	30.1	37	19.9	51	27.4	42	22.6	0.210
Yes	181	100	52	28.7	47	26.0	35	19.3	47	26.0	
Trained nursing administrator for IPC											
No	136	100	46	33.8	32	23.5	23	16.9	35	25.7	0.125
Yes	234	100	62	26.5	52	22.2	64	27.4	56	23.9	
Training staff for infection prevention											
No	118	100	39	33.1	27	22.9	26	22.0	26	22.0	0.680
Yes	252	100	69	27.4	57	22.6	61	24.2	65	25.8	
Evaluation of hand hygiene compliance among nursing staff											
No	280	100	84	30.0	61	21.8	67	23.9	68	24.3	0.846
Yes	90	100	24	26.7	23	25.6	20	22.2	23	25.6	
Provision of portable alcohol hand sanitizer to staff											
No	28	100	12	42.9	7	25.0	6	21.4	3	10.7	0.218
Yes	342	100	96	28.1	77	22.5	81	23.7	88	25.7	
Changing diapers with disposable gloves (always)											
No	29	100	6	20.7	4	13.8	8	27.6	11	37.9	0.224
Yes	341	100	102	29.9	80	23.5	79	23.2	80	23.5	
Changing diapers while wearing disposable aprons (always)											
No	299	100	90	30.1	64	21.4	72	24.1	73	24.4	0.608
Yes	71	100	18	25.4	20	28.2	15	21.1	18	25.4	
Monitoring for results of vaccination and antibody titer test for staff											
No	200	100	67	33.5	51	25.5	41	20.5	41	20.5	0.030
Yes	170	100	41	24.1	33	19.4	46	27.1	50	29.4	

Chi‐squared test or Fishers' exact tests were conducted. 1st quartile: 2.5–3.0 full‐time equivalent nurses; 2nd quartile: 3.0–4.0; 3rd quartile: 4.0–5.8; and 4th quartile: 5.8–28.3.

IPC, infection prevention and control.

In the univariate logistic regression models, following a manual for infection prevention (odds ratio [OR] 2.19, 95% confidence interval [CI] 1.07–4.47, *P* < 0.05), having a committee for IPC (OR 2.29, 95% CI 1.20–4.38, *P* < 0.05) and training staff for infection prevention (OR 1.87, 95% CI 1.17–2.97, *P* < 0.01) were significantly associated with the incidence of infection (refer to Tables 3 and S2). After adjusting for home‐care nursing agency characteristics (i.e., agency ownership, agency with medical institution, number of full‐time equivalent nurses, percentage of users with care need level ≥3, percentage of users under medical treatment), the following associations were still statistically significant: having a committee for IPC (OR 2.19, 95% CI 1.11–4.34, *P* < 0.05) and training staff for infection prevention (OR 1.67, 95% CI 1.02–2.72, *P* < 0.01), as well as being significantly associated with the incidence of infection (refer to Table 3).

**Table 3:** Results of logistic regression models for IPC practice and incidence of infection (*N* = 370)

	Univariate regression models				Multivariate regression models^†^			
	OR	95% CI		*P*‐value	Adjusted OR	95% CI		*P*‐value	
Having a manual for infection prevention	2.19	1.07	4.47	<0.05	2.03	0.95	4.36	0.068
Having a committee for infection prevention	2.29	1.20	4.38	<0.05	2.19	1.11	4.34	<0.05
One assigned representative as infection control professional	1.11	0.65	1.89	0.715	1.02	0.57	1.81	0.953
Exchange the information regarding IPC with other agencies	1.09	0.70	1.71	0.692	1.05	0.66	1.68	0.827
Trained for IPC for nursing administrator	1.14	0.72	1.80	0.564	0.90	0.55	1.47	0.669
Training for infection prevention for staff	1.87	1.17	2.97	<0.01	1.67	1.02	2.72	<0.05
Evaluation for hand hygiene compliance among nursing staff	1.03	0.62	1.74	0.898	1.02	0.58	1.77	0.954
Provision of portable alcohol hand sanitizer to staff	0.90	0.39	2.12	0.814	0.78	0.32	1.92	0.589
Changing diapers with disposable groves (always)	1.03	0.45	2.33	0.952	0.76	0.32	1.82	0.542
Changing diapers while wearing disposable aprons (always)	0.83	0.48	1.44	0.507	0.81	0.45	1.47	0.493
Monitoring for results of vaccination and antibody titer test for staff	0.95	0.61	1.47	0.807	0.69	0.42	1.14	0.150

CI, confidence interval; IPC, infection prevention and control.

^†^ Independent variable was each items of infection prevention and control practice. Also adjusted for agency ownership, agency with medical institution, number of full‐time equivalent nurses, percentage of users with care need level ≥3, percentage of users who are under medical treatment.

## Discussion

To our knowledge, this study is the first to demonstrate nationwide IPC practices of home‐care nursing agencies in Japan. We found that adherence to IPC practices varied across contents and agencies, which is consistent with previous studies.^2^, ^10^ We also identified challenges associated with IPC policy and administrative structure, and adherence to standard precautions. Almost all agencies have a manual for IPC; hence, agencies with committees for IPC or with one assigned representative as an infection control professional were a minority. Small agencies were less likely to ensure the presence of a manual or institute a committee for IPC.

Although portable alcohol hand sanitizers were available in almost all agencies, only a few agencies assessed the nursing staff's compliance with hand hygiene practices. Notably, a difference between self‐reported compliance with hand hygiene practices and observed compliance was apparent.^11^ In addition to the distribution of resources, actual compliance must be monitored by agencies. As for standard precautions, the use of gloves was common, but the use of aprons as PPEs during diaper change was rare. The WHO recommends that gloves and gowns should always be worn when providing care for patients at a community setting, regardless of the patient's diagnosis, to reduce the risk of infection.^28^ Concerning the use of PPEs, the financial burden of disposable PPEs is validated in long‐term care facilities.^29^ It can be assumed that the burden is even greater for home healthcare agencies, which are smaller and have a weaker management base. In Japan, the Long‐term Care Act (Article 70, 115‐2), an ordinance of the Ministry of Health, Labour and Welfare (No. 37; March 3, 1999), requires all home‐care nursing agencies to be equipped with the necessary equipment and devices to deliver home‐care nursing services. In addition to this national mandate, most local governments set their own requirements, in which home‐care nursing agencies need to be equipped with hand hygiene equipment to prevent infection. Although there is a financial incentive for patient safety management named “fee for management of home‐care nursing” in the fee schedule in the insurance system,^30^ it does not cover the cost of PPEs, and therefore each home‐care nursing agency must purchase these by themselves. It is noteworthy, however, that policies assisting home‐care nursing agencies can provide sufficient PPEs.

We also discovered that, contrary to common belief, infection was more likely to be reported in agencies with well‐organized policy and education systems for IPC, after adjusting for home‐care nursing agency characteristics such as size and patients' care and medical treatment requirement levels. This correlation might be explained by the fact that well‐organized agencies are more likely to establish a surveillance system that can accurately detect infections in patients. Home‐care nursing agencies with patients who are at a high degree of care need level and under medical treatment need to be mindful of the incidence of infection and those patients' needs. Valid and standardized surveillance systems have been an issue among IPC in home‐care settings.^1^, ^8^, ^21^ To obtain the fee for management of home‐care nursing in Japan, the establishment of patient safety management and a reporting system at the agency is required, but no further elaboration is provided.^30^ More valid and evidence‐based guidelines regarding the establishment of a surveillance system for IPC at home‐care settings should be available for all agencies.

This study has some limitations. First, we used self‐reported items from the questionnaire. Consequently, adherence to IPC practice may have been overreported, and the incidence of infection might have been underreported. Further studies using observational surveys or validated surveillance systems are required. Second, the survey had a low response rate of 3.7%, because the COVID‐19 outbreak occurred during the study period. Similarly, agencies demonstrating good practices were more likely to respond to the survey. Nevertheless, the responses were equally derived from all regions across the nation^24^ and the characteristics concerning agency ownership and size were not different from the nationwide statics (refer to Table S1). Hence, this study can describe the nationwide situation. Third, as this study was carried out in March 2020, which was just as the COVID‐19 outbreak began in Japan, the findings of this study may differ from the current situation under the COVID‐19 outbreak. Nevertheless, this study highlights the nationwide situation of IPC among home‐care nursing agencies before the COVID‐19 outbreak. Based on the findings of this study as a baseline situation, future studies can verify the current adherence to IPC practice during the COVID‐19 outbreak.

To conclude, this study depicts the nationwide situation of IPC practice across home‐care nursing agencies, outlining several challenges in the establishment of IPC policies, administrative structures and adherence to standard precautions. Besides, well‐organized agencies were found to be more likely to detect infections occurring over the past 3 months, after adjusting for home‐care nursing agency characteristics. Support needs to be available to enable agencies to ensure good IPC practices and to establish a surveillance system.

## Disclosure statement

The authors declare no conflict of interest.

## Authors' contributions

NM and MK: study concept and design; MK: data acquisition; NM and MK: data analysis; NM and MK: data interpretation; NM and MK: manuscript preparation; NM and MK: critical revision of the manuscript.

## Supporting information


**Table S1** Comparison among final study sample, study participants and national 1 statistics.

**Table S2** Result of univariate logistic regression models for IPC practice and incidence of infection (*n* = 370).
