# Supplementary material for: Infection prevention and control practice among home‐care nursing agencies in Japan: Secondary analysis of a nationwide cross‐sectional survey
Source: Geriatr Gerontol Int. 2021 Sep 1;21(10):913–8. doi: 10.1111/ggi.14266 (PMC9292936; doi:10.1111/ggi.14266)
Supplement: Supplementary file 1 — Table S1 Comparison among final study sample, study participants and national 1 statistics. [file GGI-21-913-s001.pdf]

Table S1. Comparison among final study sample, study participants and national statistics.

	Final study sample		Study participants §		Survey of Institutions and Establishments for Long-term Care in 2018¶	
	n=370		n=496		n=10884	
Region (n, %)						
Hokkaido	23	6.2	29	5.8	492	4.5
Tohoku	16	4.3	22	4.4	619	5.7
Kanto	99	26.8	125	25.2	2967	27.3
Chubu	54	14.6	67	13.5	1704	15.7
Kinki	81	21.9	110	22.2	2576	23.7
Shikoku・Chugoku	39	10.5	51	10.3	1082	9.9
Kyusyu・Okinawa	57	15.4	73	14.7	1444	13.3
Missing	1	0.3	19	3.8	0	0.0
Agency ownership (n, %)						
Healthcare corporation	111	30.0	141	28.4	2802	25.7
Profit	159	43.0	206	41.5	5476	50.3
Social welfare	80	21.6	105	21.2	2141	19.7
Others	20	5.4	29	5.8	223	2.0
Missing	0	0.0	15	3.0	242	2.2
Number of nurses (full-time equivalent) (mean, standard deviation)	4.8	2.7	4.8	2.7	5.3	N. A

§ We excluded 84 questionnaires from 580 questionnaires that were returned because those were without agree for study participation

¶ Survey of Institutions and Establishments for Long-term Care was conducted by the Ministry of Health, Labour and Welfare, and targeted all of the institutions and establishments in the Long-Term Care Insurance system in Japan
