# Supplementary material for: Infection prevention and control practice among home‐care nursing agencies in Japan: Secondary analysis of a nationwide cross‐sectional survey
Source: Geriatr Gerontol Int. 2021 Sep 1;21(10):913–8. doi: 10.1111/ggi.14266 (PMC9292936; doi:10.1111/ggi.14266)
Supplement: Supplementary file 2 — Table S2 Result of univariate logistic regression models for IPC practice and incidence of infection (n = 370). [file GGI-21-913-s002.docx]

Table S2. Result of univariate logistic regression models for IPC practice and incidence of infection (n=370).

|  | | | Odds Ratio | 95% CI | | | | | p-value | | |  |
| --- | --- | --- | --- | --- | --- | --- | --- | --- | --- | --- | --- | --- |
| Infection prevention and control practice | | |  | |  | |  | | |  | | |
|  | Having a manual for infection prevention | | 2.19 | | 1.07 | | 4.47 | | | <0.05 | | |
|  | Having a committee for infection prevention | | 2.29 | | 1.20 | | 4.38 | | | <0.05 | | |
|  | A representative assigned as an infection control professional | | 1.11 | | 0.65 | | 1.89 | | | 0.715 | | |
|  | Exchanging information regarding IPC with other agencies | | 1.09 | | 0.70 | | 1.71 | | | 0.692 | | |
|  | Training nursing administrator for IPC | | 1.14 | | 0.72 | | 1.80 | | | 0.564 | | |
|  | Training staff for infection prevention | | 1.87 | | 1.17 | | 2.97 | | | <0.01 | | |
|  | Evaluating hand hygiene compliance among nursing staff | | 0.90 | | 0.39 | | 2.12 | | | 0.814 | | |
|  | Provision of portable alcohol hand sanitizer to staff | | 1.03 | | 0.62 | | 1.74 | | | 0.898 | | |
|  | Changing diapers with disposable gloves (always) | | 1.03 | | 0.45 | | 2.33 | | | 0.952 | | |
|  | Changing diapers while wearing disposable aprons (always) | | 0.83 | | 0.48 | | 1.44 | | | 0.507 | | |
|  | Monitoring results of vaccination and antibody titer test for staff | | 0.95 | | 0.61 | | 1.47 | | | 0.807 | | |
| Agency characteristics | | |  | |  | |  | | |  | | |
|  | Years since establishment (ref. 1^st^ quartile) | |  | |  | |  | | |  | | |
|  | 2nd quartile | | 0.94 | | 0.53 | | 1.66 | | | 0.829 | | |
|  | 3rd quartile | | 1.15 | | 0.58 | | 2.26 | | | 0.691 | | |
|  | 4th quartile | | 1.23 | | 0.65 | | 2.33 | | | 0.524 | | |
|  | Agency ownership (ref. healthcare corporation) | |  | |  | |  | | |  | | |
|  | Profit | | 0.43 | | 0.25 | | 0.75 | | | <0.01 | | |
|  | Social welfare | | 0.83 | | 0.42 | | 1.63 | | | 0.585 | | |
|  | Others | | 0.51 | | 0.18 | | 1.43 | | | 0.200 | | |
|  | Agencies with a medical institution (ref. without) | | 1.83 | | 1.13 | | 2.96 | | | <0.05 | | |
|  | Number of full-time equivalent nurses (ref. 1st quartile) |  |  | | |  | | | | |  |  |
|  | 2nd quartile | | 1.23 | | 0.67 | | 2.27 | | | 0.509 | | |
|  | 3rd quartile | | 1.54 | | 0.82 | | 2.88 | | | 0.176 | | |
|  | 4th quartile | | 1.11 | | 0.62 | | 2.02 | | | 0.721 | | |
|  | Agency with an advance practice nurse (ref. without) | | 1.19 | | 0.62 | | 2.30 | | | 0.606 | | |
|  | Number of users in a month (ref. 1st quartile) | |  | |  | |  | | |  | | |
|  | 2nd quartile | | 2.03 | | 1.09 | | 3.80 | | | <0.05 | | |
|  | 3rd quartile | | 1.86 | | 0.99 | | 3.50 | | | 0.053 | | |
|  | 4th quartile | | 1.34 | | 0.74 | | 2.45 | | | 0.338 | | |
|  | Nurse managers' years of experience as a manager | | 1.03 | | 0.97 | | 1.09 | | | 0.306 | | |
|  | Accept pediatric patient (ref. without) | | 0.95 | | 0.56 | | 1.62 | | | 0.860 | | |
|  | Accept patient at terminal care stage (ref. without) | | 1.03 | | 0.63 | | 1.69 | | | 0.908 | | |
|  | Percentage of users with a care need level ≧3 (ref. 1^st^ quartile) | |  | | |  | | | | |  |  |
|  | 2^nd^ quartile | | 2.05 | | 1.11 | | 3.80 | | | <0.05 | | |
|  | 3^rd^ quartile | | 2.30 | | 1.23 | | 4.29 | | | <0.01 | | |
|  | 4^th^ quartile | | 1.99 | | 1.08 | | 3.69 | | | <0.05 | | |
|  | Percentage of users who needs medical treatment (ref. 1^st^ quartile) | | | | | | |  |  |  |  |  |
|  | 2^nd^ quartile | | 1.78 | | 0.97 | | 3.25 | | | 0.061 | | |
|  | 3^rd^ quartile | | 2.23 | | 1.19 | | 4.17 | | | <0.05 | | |
|  | 4^th^ quartile | | 2.40 | | 1.28 | | 4.51 | | | <0.01 | | |

IPC: Infection prevention and control; CI: Confidence interval
